# Preliminary characterization of IL32 in basal-like/triple negative compared to other types of breast cell lines and tissues

**DOI:** 10.1186/1756-0500-7-501

**Published:** 2014-08-07

**Authors:** Audrey Player, Tim Oguamanam, Jennifer Okanmelu, Kayla Burrell, Mario Hollomon

**Affiliations:** 1 The Department of Biology, Texas Southern University, Houston, Texas 77004, USA

**Keywords:** Triple negative, Basal breast cancers, Gene expression analysis, IL32

## Abstract

**Background:**

Triple negative breast cancer (TNBC) and often basal-like cancers are defined as negative for estrogen receptor, progesterone receptor and Her2 gene expression. Over the past few years an incredible amount of data has been generated defining the molecular characteristics of both cancers. The aim of these studies is to better understand the cancers and identify genes and molecular pathways that might be useful as targeted therapies. In an attempt to contribute to the understanding of basal-like/TNBC, we examined the Gene Expression Omnibus (GEO) public datasets in search of genes that might define basal-like/TNBC. The Il32 gene was identified as a candidate.

**Findings:**

Analysis of several GEO datasets showed differential expression of IL32 in patient samples previously designated as basal and/or TNBC compared to normal and luminal breast samples. As validation of the GEO results, RNA and protein expression levels were examined using MCF7 and MDA MB231 cell lines and tissue microarrays (TMAs). IL32 gene expression levels were higher in MDA MB231 compared to MCF7. Analysis of TMAs showed 42% of TNBC tissues and 25% of the non-TNBC were positive for IL32, while non-malignant patient samples and all but one hyperplastic tissue sample demonstrated lower levels of IL32 protein expression.

**Conclusion:**

Data obtained from several publically available GEO datasets showed overexpression of IL32 gene in basal-like/TNBC samples compared to normal and luminal samples. In support of these data, analysis of TMA clinical samples demonstrated a particular pattern of IL32 differential expression. Considered together, these data suggest IL32 is a candidate suitable for further study.

## Findings

TNBC are defined as negative for expression of three genes, estrogen receptor (ESR), progesterone receptor (PR) and Her2 gene, and account for 15-20% of breast cancer types. They represent a sub-population of breast cancers that demonstrate aggressive behavior, overall poorer prognosis, and higher grade and risk of recurrence compared to other types of breast cancers [1]. Basal-like breast cancers are also aggressive, with overall poorer prognosis, negative for ESR/PR/Her2 and positive for HER1+ and cytokeratin 5/6+ [2,3]. Often times investigators refer to basal-like and TNBC as synonymous type of cancer. Even though basal-like cancers and TNBC are similar, data show that on a molecular level, they are somewhat different. Prat et al. [4] performed microarray analyses of 412 TNBC and 473 basal-like samples and showed that 21.4% of the TNBC were non-basal-like, and 31.5% of the basal-like tumors were non-TNBC. In another study Lehmann et al. [5] identified seven subtypes within TNBC samples, including basal1, basal2, immunomodulatory, mesenchymal, mesenchymal stem-like, luminal androgen receptor and an unstable type. Rody et al. [6] analyzed samples that were previously characterized as TNBC and found that 73% of TNBC demonstrated a basal-like molecular signature while the remaining samples were not basal-like. Collectively, these data are evidence that samples characterized as TNBC consist of a subpopulation of samples with diverse molecular characteristics, those which clearly align with basal-like signatures, and those displaying characteristics similar to other breast subtypes. These studies emphasize the differences between TNBC and basal-like cancers, but overwhelmingly the data also show the similarities between the cancers and reveal the molecular complexities of breast cancers in general.

From a therapeutic standpoint, it is critically important that we understand TNBC and basal-like cancers. While effective targeted gene therapies exist for breast cancers displaying positive receptor status, fewer treatment options exist for patients displaying negative receptor status. Breast cancers displaying positive receptor status are effectively treated with hormonal therapies that target their receptor genes and Her2. Effective molecular targets related to basal-like/TNBC cancers have not been identified. Effective therapeutic targets can only come from understanding the biology of the disease and identifying reliable genetic signatures to study for their potential use in treating patients. The development of new, specific targeted therapies will be of significant benefit to patients unresponsive to current therapies. In an attempt to contribute to these studies, we examined publically available GEO datasets in search of novel genes that might be differentially expressed in breast cancers. Taking into account the similarity between the two cancers, we specifically chose to analyze public datasets that were previously characterized as basal-like/TNBC, luminal and non-tumor in search of genes that were differentially expressed in both the basal-like and TNBC subtypes compared to the other subtypes. For our initial selection, datasets were limited to those with online data-analyses tools and because of the similarity between basal-like and TNBC, datasets were selected based on search filters using the terms “basal breast cancer”. From our search, we identified datasets containing patient samples and cell lines in which the receptor status was known. The datasets were analyzed and the candidate genes were selected. The IL32 gene was selected for further study because it was differentially expressed in basal-like clinical samples and TNBC cell lines, compared to samples defined by the other subtypes.

The IL32 gene was initially identified by Kim et al. [7] following overexpression of IL18 receptor-beta in A549 human lung adenocarcinoma cells. The IL32 gene is a pro-inflammatory cytokine produced by immune, endothelial and epithelial cell types and is processed as alternative splice variants. Limited data exist describing the IL32 cytokine in cancers; however there is evidence the gene is suppressive in some cancers and facilitative in other cancers. IL32 was shown to suppress hepatocellular cancers, melanoma and colon cancers [8]. Oppositely, overexpression of IL32 has been shown to be an independent prognostic marker for gastric cancer [9,10].

As part of this study, we observed overexpression of IL32 gene in a subgroup of the basal-like/TNBC samples compared to normal and luminal samples retrieved from GEO. We then experimentally validated IL32 RNA and protein expression levels in cell lines and tissue microarrays (TMAs) in which the receptor status was known; a similar pattern of differential expression was observed. To our knowledge this is the first study demonstrating a differential pattern of IL32 expression in breast tissues.

## Methods

### Cell culture and tissue microarray samples

MCF7 cells were used to represent non-basal-like (receptor positive) samples and MDA MB231 cell line was used to represent TNBC/basal-like samples. Cell lines were purchased from American Type Culture Collection (ATCC; Manassas VA). MCF7 and MDA-MB-231 cell lines were grown in DMEM/F12 (Gibco/Invitrogen; Carlsbad, CA) culture media, supplemented with 10% fetal bovine serum and an antimycotic solution. All of the cells were maintained in a 37°C incubator with 5% CO_2_ in a humidified environment. For analysis of patient samples, paraffin-embedded tissue microarray (TMA; catalog # BRC1506 and BRC1021) samples were purchased from Pantomics Incorporated (Richmond CA). TMAs included different breast tissues representing an assortment of clinical diagnoses. Documents supplied by the company state that they are dedicated to the ethical procurement of tissues from human donors and the ethical treatment of animals, and all tissues are collected under strict guidelines and consent. TMA samples were supplied with age, pathology, grade, stage, ESR, PR and Her2 status. The ESR/PR/Her2 receptor status of each TMA was determined by immunohistochemistry (IHC) staining and scored by pathologists affiliated with the company. Scores ranged from 0–3; samples designated as “0” for all three receptors were designated as TNBC, and values higher than zero were considered positive for one or more receptors. Tissue samples that were positive for 1 or more receptor were considered non-TNBC in this study.

### Data analysis

The breast cancer datasets were selected from GEO [11]. Datasets were initially selected using the search terms “Affymetrix/basal/breast cancer”. One hundred thirty seven GEO datasets appeared using these terms. We examined each dataset and screened for (a) samples processed using the Affymetrix U133 microarray platform (Santa Clara CA; [12-14] and (b) human/breast cancer samples that were not genetically manipulated or previously treated with drugs. Eight datasets were selected but 3 were excluded because they either contained too few breast samples or in the case of one, the dataset contained only TNBC samples. The five datasets included GDS2250, GDS1329, GSE34211, GSE7904 and GSE12777. We later discovered that GDS2250 and GSE7904 were separate studies originating from the same clinical sample dataset. GDS2250 and GDS1329 datasets included clinical patient samples and GSE34211 and GSE12777 datasets included studies using cell line samples.

For our initial analyses, preference was given to datasets designated by GEO as “curated”, because the curated datasets included online tools that allowed for T-test comparisons, gene cluster displays, an up-down selection function to display differentially expressed genes, normalized individual gene-expression-profile charts and neighbor-analyses functions. The GDS2250 and GDS1329 are curated datasets. Since we were able to use the online analysis tools available for the curated samples, GDS2250 and the GDS1329 datasets were processed first. The remaining 2 datasets were used to assess gene expression levels in datasets containing basal-like/TNBC samples.

The GDS2250 and GDS1329 datasets contained different types of samples. Samples in the GDS2250 dataset were previously identified as normal, non-basal-like and basal-like breast cancer types; samples in the GDS1329 dataset were previously identified as apocrine, basal and luminal type breast cancers. Throughout our analyses, we maintained the nomenclature assigned to the dataset; for example if samples were designated non-basal-like (as in GDS2250), we continued that designation throughout our study. Samples in the GDS2250 dataset were designated as normal, non-basal-like and basal-like, as a result, our approach was to focus on genes differentially expressed in the basal-like samples compared to normal and non-basal-like. Initially the goal of our studies was to identify genes differentially expressed in TNBC, however, recognizing the relationship between basal-like and TNBC, we focused on identifying samples related to both subtypes.

The GDS2250 and GDS1329 datasets were analyzed separately in search of differentially expressed genes, with GDS2250 analyzed first. The GDS2250 datasets were examined using GEO’s “Data Analysis tool” and the linear relationship was established using Pearson distance correlation. Hierarchical cluster and gene expression patterns were examined and particular gene clusters were selected. Individual gene expression profiles related to the dataset were validated by searching the “GEO gene-expression-profile charts” using keywords “GDS2250 and the gene-of-interest”. GDS1329 was examined following the same approach. Gene-expression-profile charts available in GEO were used to validate the expression patterns. Differentially expressed genes common between GDS2250 and GDS1329 were selected as possible gene candidates. Based on the initial selection methods, candidates were defined as basal-like genetic signatures. IL32 and several other genes were identified as differentially expressed following analysis of the GDS2250 and GDS1329 datasets.

Candidate genes were further examined for expression in the GSE34211 and GSE12777 datasets. The GSE34211 and GSE12777 datasets contained cell lines characterized as luminal and basal-like. The receptor status of the cell lines was known which allowed for analysis of IL32 in TNBC samples. Gene-expression-profile charts were not available at GEO for the two datasets, however normalized gene expression values were available. IL32 was finally selected because the gene showed differential expression in GDS2250, GDS1329, in several TNBC cell lines examined as part of the GSE34211 study and in the GSE12777 dataset. For direct comparison across datasets, the IL32 Affymetrix 203828_s_at probe-set was analyzed for all of the samples examined.

Once the IL32 gene was identified, the gene expression values corresponding to the Affymetrix 203828_s_at probe-set were downloaded into Excel. Gene expression values related to individual datasets were listed as either normalized signal intensities or log2 values. For the datasets in which IL32 was listed as log2 values, for comparison across the four different datasets, the values were converted back to the normalized signal intensity value using the Excel antilog function.

Data show that IL32 gene is processed as alternative slice variants. The sequences corresponding to the IL32 splice variants were retrieved from Ensembl [15], NCBI Reference Sequences (RefSeq) [16] and GeneCards [17], and the gene alignments were performed using the Multi-Align™ program [18].

### cDNA generation and real-time PCR validation

For each sample, one microgram of total RNA was used to generate cDNA using the iScript kit (BioRad Life Science; Hercules, CA). The cDNA samples were diluted to 10 ng/ul with RNAse free water and stored at -20°C until ready for PCR. The gene primer-sets were designed using Primer3 [19]. Primer-sets were synthesized by IDTDNA (Coralville, IA). The IL32 gene exists as spliced transcript variants. We chose to use IL32 RefSeqs as our resource for the IL32 gene. We identified eight reference sequence (RefSeq) transcript variants deposited at NCBI [16] and GeneCards [17]. The sequences were retrieved and analyzed using tools available at the University of California at Santa Cruz (UCSC) Genome Browser [20]. The final set of primers used for the study was designed to accomplish two goals. To begin, IL32 primers were designed to recognize as many of the RefSeqs as possible (Additional file 1: Table S1). Then, primers were designed to recognize the same antigenic region as recognized by the antibody used for our study, allowing for direct comparisons between results observed for the RNA and protein studies. The experimental approach related to the protein studies is described below. Related to the RNA studies, several different primer-sets were tested, but ultimately we choose IL32 primers which included the forward primer sequence AGCTGGAGGACGACTTCAAA and the reverse primer sequence CCTGGAACCATCTCATGACC, which generated a 207 base pair (bp) amplicon. The Mitochondrial ribosomal protein L19 (MRPL19) gene was used as the endogenous control which included a forward primer sequence of AAGCGTCGAAAAGGTCTTGA and reverse sequence of GCTCAGGTTCCATGCTCATT which generated a 306 bp amplicon. The specificity of the primer-sets was validated using the web-based *in-silico* PCR program available as a part of the UCSC Genome Browser [20]. PCR reactions were performed using the Bio-Rad PCR amplification system.

### Western blotting and immunohistochemistry

Cluster of differentiation 1 (CD31) antibody (ab133191) was purchased from Abcam (Cambridge, MA), and the IL32 antibody (NBP-1 82560) was purchased from Novus Biologicals (Littleton, CO). The IL32 antibody recognizes the FPKVLSDDMKKLKARMHQAIERFYDKMQNAESGRGQVMSSLAELEDDFKEGYLETVAAYYEEQHPELTPLLEKERDGLRCRTMA sequence, corresponding to IL32 specific protein and transcript sequences [21]. We found that investigators involved in generating the IL32 antibody used Ensembl database as their source for the IL32 sequences instead of NCBI. The IL32 antibody supplied by Novus Biologicals has been used to examine an assortment of different tissues and diseases as part of the Human Protein Atlas project [22]. Related to their study, the Protein Atlas project provides (a) a list of the IL32 variant sequences that match the antigen sequence and (b) a direct link for convenient retrieval of the matching variant sequences, available through the Ensembl database. The protein and transcript variant identifications for the sequences that match the antigenic region (i.e., epitope) are listed in Additional file 2: Table S2 [15,21]. Only sequences that match 100% of the epitope are listed in the table.

Our goal was to design a PCR primer-set to detect all or most of the sequences recognized by the commercial IL32 antibody and the IL32 RefSeqs. Once accomplished, the Multialign™ program was used to determine the similarity between the sequences. In summary, the Multialign™ comparisons demonstrate the similarity between (a) the transcripts corresponding to the IL32 protein sequences used to generate the antibody (b) the RefSeq transcripts corresponding to the IL32 sequences used to generate the PCR amplicon and (c) the sequence corresponding to our 207 bp IL32 PCR amplicon (Additional file 3: Figure S1). For this particular alignment, the approximate location of the amplicon is between nucleotides 576–783. Results show that our PCR primers recognize most of the RefSeqs and transcripts defined as matching to the protein epitope.

The same antibody was used for Western blotting and IHC studies. Western blotting was performed as suggested by Novus Biologicals. As control for the western blotting, an IL32 protein lysate (NBL1-11952) was purchased from Novus Biologicals. The lysate was generated from HEK293T cells that were transfected with a plasmid containing the IL32 gene insert (NM_004221). The empty vector was supplied with the IL32 lysate, and served as the negative control for IL32 expression. Positive and negative IL32 controls were loaded at equivalent concentrations and only exposed long enough for detection of IL32 positive control. Actin gene was used as the endogenous control gene. IHC was performed as recommended by Vector Labs (Burlingame, CA).

TMA samples were deparaffinized and antigen retrieval performed by heating the TMAs at 98°F degrees for 45 minutes in 0.01 M sodium citrate buffer, pH 6.0. The TMAs were developed using DAB Peroxidase Substrate Kit,

3, 3’-diaminobenzidine (VectorLabs; Burlingame CA), counterstained, cover-slipped and examined. The TMAs were scored based on ranking the IHC signal intensities from 0-5+; 0 represented no-detectable signal while 5+ represented an intense signal detected on either the entire TMA core or at least 25% of an individual core. Only TMA cores that demonstrated at least a “3+” signal for the CD31 control, minus background were included in the analyses. The CD31 antibody recognizes cell adhesion molecules predominantly associated with endothelial cells. As a result, positive CD31 signal in regions other than blood vessels was considered background staining. Analysis of the IHC results was performed using the Nikon Eclipse™ microscope (Nikon Instruments Inc., Melville, NY).

## Results

### Identification and assessment of IL32 transcript(s) differentially expressed in basal-like / TNBC compared to non-basal-like samples

#### *Selection of IL32 based on gene expression in publically available GEO datasets*

**Figure 1:**
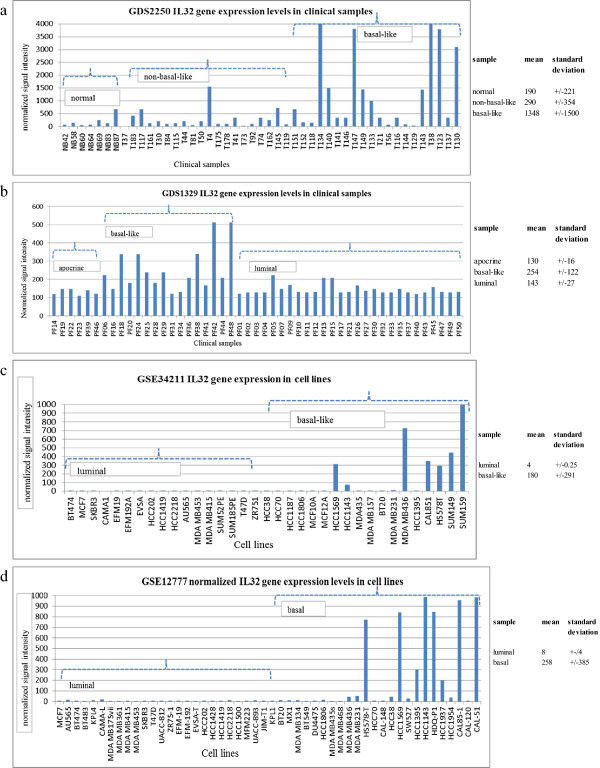
**Gene expression profiles demonstrating IL32 levels in the GEO datasets used in the study.** The plot represents the normalized IL32 gene expression levels observed for the Affymetrix 203826_s_at probeset in the **(a)** GDS2250 clinical samples **(b)** GDS1329 clinical samples **(c)** GSE34211 cell lines and **(d)** GSE12777 cell line datasets. The dashed lines are used to group the particular sample types. The normalized gene expression levels were averaged to demonstrate differences (i.e., mean) in IL32 levels between the various sample types. The mean and standard deviations values were also determined (using Excel).

It is clear from recent reports that on a molecular level, breast cancers are incredibly complex. Although defined by different subtypes, our goal was to identify genes differentially expressed in cancerous breast tissues defined as both basal-like and TNBC. The genes were initially selected based on analyses of the publically available GEO datasets. Table 1 lists the GEO datasets used for our study. Four different datasets were ultimately chosen using the criteria listed in the Methods section. Two of the datasets included clinical patient samples and two others included cell line samples. The references describing the original scientific studies are given in the table. Throughout this study we maintained the nomenclature assigned to the original dataset. If the samples are designated ‘non-basal-like’ as opposed to luminal subtype, samples are designated as non-basal when referenced in this report.We first selected genes differentially expressed in basal-like patient samples, finally selecting genes demonstrating concordant expression in TNBC cell lines. The “data analysis tools” available on GEO were used to analyze the GDS2250 breast cancer dataset and IL32 was found differentially expressed in the basal-like patient samples. GDS1329 was also examined for IL32 gene expression levels and found differentially expressed in the basal-like patient samples. There were substantial ranges in the IL32 gene expression levels between individual samples, still the mean-values for the basal-like samples were higher than values observed for the other subtypes in both GDS2250 and GDS1329 (Figure 1a and b). As validation of IL32 levels in samples of known receptor status, the GSE34211 dataset was examined. The dataset included cell line samples with positive receptors and TNBC (Figure 1c), grouped as luminal and basal-like. Only the basal-like/TNBC samples demonstrated high IL32 expression. Compared to all other samples in the dataset, at least 7 of the 18 basal-like/TNBC samples expressed high levels of IL32; MCF10A and MCF12A (non-cancer) triple negative cell lines were included in the 11 samples that expressed low levels of IL32. The GSE12777 dataset also showed higher IL32 levels in basal compared to luminal cell lines (Figure 1d). Eleven basal-like/TNBC cell lines were common between the GSE34211 and GSE12777 datasets. Of these cell lines, five (i.e., HCC1569, HCC1143, MDA MB436, Cal851 and Hs578T) displayed overexpression of IL32 in both GSE34211 and GSE12777 datasets. The relationship between these five cell lines is not obviously apparent.

**Table 1:** Summary of the publically available GEO datasets used in the study

**Gene expression omnibus**	**Type samples**	**Characteristics\subtype**	**Number of samples (n)**	**Original publications**
**(Accession number)**				
GDS2250/GSE7904	Patients	Normal/non-basal-like/basal	n = 47	Richardson AL, Wang ZC, De Nicolo A, Lu X et al. X chromosomal abnormalities in basal-like human breast cancer. Cancer Cell 2006 Feb;9(2):121–32.
GDS1329	Patients	Apocrine/basal/luminal	n = 49	Farmer P, Bonnefoi H, Becette V, Tubiana-Hulin M et al. Identification of molecular apocrine breast tumours by microarray analysis. Oncogene 2005 Jul 7;24(29):4660–71.
GSE34211	Cell lines	Luminal/basal	n = 35	Hook KE, Garza SJ, Lira ME, Ching KA et al. An integrated genomic approach to identify predictive biomarkers of response to the aurora kinase inhibitor PF-03814735. Mol Cancer Ther 2012 Mar;11(3):710-9
GSE12777	Cell lines	Luminal/basal	n = 49	Hoeflich KP, O’Brien C, Boyd Z, Cavet G et al. In vivo antitumor activity of MEK and phosphatidylinositol 3-kinase inhibitors in basal-like breast cancer models. Clin Cancer Res 2009 Jul 15; 15(14):4649–64.

The datasets were retrieved from GEO based on search terms. The types of samples used for the particular study, the number of samples processed and the reference for the original publications are listed. The characteristics and subtype designations were assigned by the original investigators.

In GSE34211, low levels of IL32 were observed in MDA MB231, but normalized signal levels exceeded MCF7; MDA MB231 was 10 compared to MCF7 which was 4. In GSE12777, MDA MB231 was clearly overexpressed compared to luminal samples. Overall, analysis of the GEO public datasets showed overexpression of IL32 in patient samples and cell lines previously characterized as either basal-like or TNBC. All of the basal-like/TNBC samples did not show overexpression of IL32, but of the samples expressing high levels, all were basal-like/TNBC.

#### *Experimental assessment of IL32 gene expression levels*

**Figure 2:**
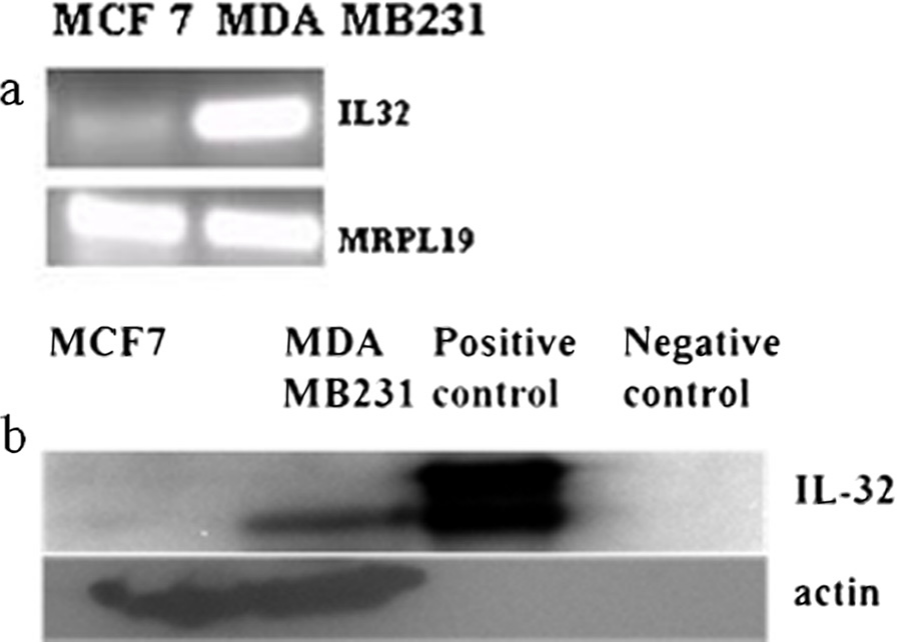
**Analysis showing overexpression of IL32 gene expression in MDA MB231 (TNBC) compared to MCF7 (receptor positive). (a)** PCR analysis of IL32 transcript in MCF7 compared to MDA MB231. MRPL19 was used as the endogenous control gene **(b)** Western analysis of IL32 protein in MCF7, MDA MB231, Positive control protein and negative control. Actin gene was used as endogenous control.

IL32 gene expression in the GEO samples was based on analysis of the Affymetrix 203826_s_at probeset. As validation of IL32 in GEO results, initially primer-sets were designed based on the same Affymetrix probe-set and differential expression of the gene assessed using MDA MB231 and MCF7 samples. However, following PCR analysis of MDA MB231 and MCF7 cDNA, results were inconsistent. Similar to the results observed in the GEO datasets, IL32 transcript levels were higher in MDA MB231 compared to MCF7, but different sized amplicons were generated depending on the cDNA preparation. The decision was then made to consider primer-sets based on different IL32 target regions. Search of the genomic databases and the Affymetrix probe-set extension (i.e., “_s_at”) indicate splice variants exist for IL32. In an attempt to generate more consistent results, primer-sets corresponding to different regions and variants were designed and tested for reproducibility. Ultimately we selected a primer-set that satisfied two criteria; the primer-set detected most of the transcript variants and primers corresponded to the same antigenic region as the antibody used for the protein studies (Additional file 3: Figure S1). After the final primer-set was chosen, IL32 levels in MCF7 and MDA MB231 were again assessed (Figure 2a). The PCR experiments were repeated more than 10 times using 3 different cDNA preparations and each time transcript levels appeared higher in MBA MB231 compared to MCF7. The experiment was also repeated using a commercial source of MCF7 total RNA and two additional endogenous control genes, all demonstrating the same results. Also, we obtained cDNA generated from MDA MB468 cell lines and examined the IL32 levels. Compared to levels observed in GSE12777, IL32 in MDA MB468 appeared lower than levels observed in MCF7 (data not shown). Consistent with PCR, western blot analysis showed that IL32 protein levels were higher in MDA MB231 compared to MCF7 (Figure 2b).

### Assessment of IL32 protein expression in tissue microarray containing clinically diagnosed patient samples

**Figure 3:**
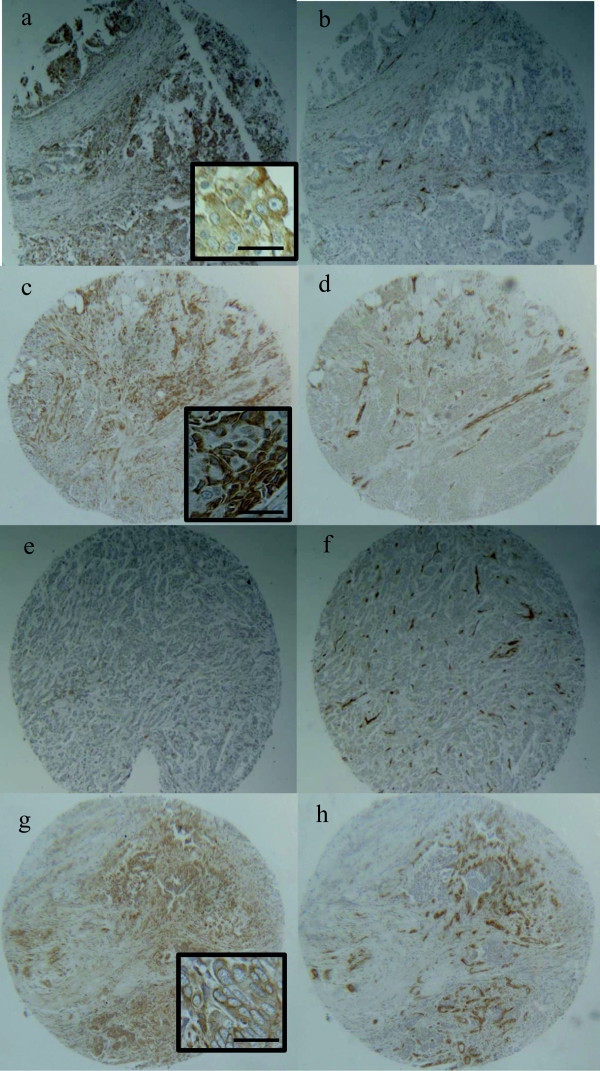
**IHC analysis of IL32 levels in TMA.** CD31 and IL32 protein expression was determined in various types of breast tissues. TNBC demonstrating **(a)** positive IL32 expression and **(b)** CD31 control expression. Non-TNBC demonstrating **(c)** positive IL32 expression and **(d)** CD31 control expression. Non-TNBC demonstrating **(e)** negative IL32 expression and **(f)** CD31 control expression. Invasive ductal or lobular carcinoma demonstrating **(g)** positive IL32 expression and **(h)** CD31 control expression. Bar = 50uM.

The same antibody used for western analysis was used for IHC analysis of the TMAs. For our studies, CD31 signal intensity was used as the control to assess the quality of the TMA tissue and the specificity of our antibody. After optimizing the antibody, if non-specific CD31 staining was observed the tissues were excluded from our analyses. Ninety four total TMA cores demonstrated CD31 signal intensities ≥ 3+, and subsequently included in the IHC analyses. A summary of the IHC results are given in Table 2. TMAs included TNBC, non-TNBC and non-malignant breast samples. The samples included on the TMAs contained clinical information and were defined based on ER/PR/Her2 status. The most substantial IL32 expression was observed in TNBC and non-TNBC. Forty-two percent of the TNBC demonstrated overexpression of IL32 while 25% of the non-TNBC demonstrated overexpression. Negligible levels of IL32 were observed in normal and all but one benign tissue. A list of the patient samples, including their clinical information and individual IL32 staining intensities are listed in Additional file 4: Table S3.Examples of the IHC results are presented in Figure 3. Figure 3a is an example of TNBC positive for IL32. Figure 3c is an example of non-TNBC positive for IL32 expression. Figure 3e is an example of a non-TNBC negative for IL32 expression. Figure 3g shows positive IL32 expression in the sample previously diagnosed as an invasive ductal carcinoma with residual normal/hyperplasia. IL32 expression in each of the samples appeared to be localized to the cytoplasm of the epithelial cells. The corresponding CD31 signal intensities for the samples noted above are shown in Figures 3b, d, f and h.

**Table 2:** Summary of IHC analysis of IL32 in patient samples (TMA)

**Pathology**	**Nature**	**Receptor status**	**# samples (Signal Intensity)**	**% with signal ≥ 3**
Normal	Normal	Non triple negative	3/9 (1–2) ; 6/9 ND	0%
Fibroadenoma	Benign	Non triple negative	0/6 (0–1)	0%
Invasive ductal carcinoma with residual normal/hyperplasia	Benign	Non triple negative	1/1 (3)	100%
Atypical/Seveous atypical hyperplasia	Hyperplasia	Non triple negative	0/4 (0–1)	0%
Ductal carcinoma/Invasive ductal or lobular carcinoma	Malignant	Non triple negative	16/64 (3–4) ; 9 (1–2); 39/64 ND	25%
Invasive ductal carcinoma	Malignant	Triple negative	5/12 (3–4) ; 7/12 ND	42%
				
				

The pathology, nature and receptor status were determined by the TMA vendor. Ninety four total cores demonstrated CD31 signal intensity ≥ 3, and subsequently included in the IHC analyses. The IL32 signal intensities were scored and the number of samples analyzed and their corresponding IL32 signal intensities listed. The percent of samples with IL32 signal intensities ≥ 3+ were also given. ND = not detected. The signal intensity determined by 2 operators (AP and TO).

Similar to the results observed in the GEO datasets, IL32 gene expression was detected in several of the TNBC and non-TNBC. Many of the TNBC and non-TNBC did not display IL32 expression, and negligible levels were observed in normal breast tissues. At this point, we have not determined the implications of IL32 expression in the malignant tissues. Future analyses will include analysis of samples from a variety of different diagnoses, including tumors with infiltrating inflammatory cell types.

## Discussion

For this current study, we observed the IL32 gene differentially expressed in basal-like and TNBC, performed preliminary analysis of gene expression in particular cell lines and tissue samples, and suggested it be examined further for its role in breast cancers. IL32 is a cytokine, and immune-related genes and cell types are proving to be important tools in understanding the contribution of the tumor’s microenvironment in breast cancer pathogenesis. It is premature to suggest such a role for IL32, but recent studies suggest that particular immune cells and genes are clinically useful as prognostic or predictive indicators for breast cancers. Rody et al. [23] identified a Lymphocyte specific kinase (LCK; T-cell specific) cluster of immune related genes that could separate the ESR-negative patient population based on better or worse prognosis. In a separate study, Denkert et al. [24] showed that lymphocyte infiltration could be used to predict a patients’ response to chemotherapeutic agents, where patients with previous infiltration responded more favorably to therapy. Also, Gu-Trantien et al. [25] found that CD4+ follicular T-cells infiltrations in breast cancers could be used to predict breast cancer survival. Unlike the studies cited above, this current study is only the first observation of IL32 differential gene expression in breast samples. A number of different validating experiments must be completed if we are to establish the gene’s possible utility. IL32 was discovered recently, so compared to other genes mentioned in this report, relatively little information exists demonstrating its role in clinical conditions.

IL32 has been shown to be involved in immune-related defense mechanisms [26]. Considering the nature of the gene, its activity has been established in inflammatory diseases such as rheumatoid arthritis [27], rhinosinusitis [28] chronic obstructive pulmonary disease [29] and inflammatory bowel disease [30]. IL32 levels showed positive correlations with tumor necrosis factor alpha, ESR, C-reactive protein, replication factor, and Disease Activity Score 28 genes in rheumatoid arthritis [31]. Studies related to mechanisms show that IL32 is induced by NF-kappa-B [32] and in T cells and keratinocytes it might play a role in apoptosis induced by T cell receptor signaling and Interferon gamma γ [33,34]. Other studies show the gene might regulate other cytokines [35] and affect cell adhesion [36]. All of the mechanisms attributed to IL32 could to some degree affect tumor progression.

The role of IL32 has also been examined in cancers, supporting the myriad of data supporting a relationship between inflammation and cancer. IL32 has been shown to support tumor suppression in transgenic mice inoculated with melanoma and colon cancer cells [37] and up-regulated in lung adenocarcinomas [38] where it is considered a potential clinical target. In lung adenocarcinomas, IL32 is highly expressed in both tumor cells and infiltrating leukocytes leading investigators to suggest that both the lung tumor cells and tumor infiltrating leukocytes are involved in tumor progression. IL32 is a cytokine and cytokines have also been implicated in breast cancer pathogenesis. Hartman et al. [39] show that blocking the pro-inflammatory cytokine IL6 or associated pathways might be a useful therapeutic strategy for TNBC supporting a role of cytokines in the tumorigenic process. Other studies show high levels of IL6 in serum of breast cancers appear to be related to poor prognosis [40], possibly involved in epithelial-to-mesenchymal transition [41]. Barclay et al. [42] showed that overexpression of Suppressor of Cytokine Signaling 3 molecules (SOCS3) suppresses STAT3 expression, which then decreases cell proliferation of breast cancer cells. These data suggest SOCS3 might have a role in tumor progression.

We report IL32 levels in breast cancer cells and clinical samples in which the receptor status and diagnosis has been previously determined. Our study is meant to be a preliminary analysis of IL32, demonstrating its expression in breast tissues. We observed IL32 overexpression in select TNBC, non-TNBC, and a hyperplastic breast sample compared to normal breast tissues, but we have not determined the implications of IL32 expression. A main objective of this study remains identification of genes that distinguish TNBC so they might be considered for future studies. To better understand the role of IL32 in breast tissues, the individual splice variants and coding proteins must be examined, current results must be validated using samples with more detailed clinical information, knock-down studies should be performed, and signaling and receptor proteins should be identified to determine the contribution of the gene to tumor pathogenesis. None of these experiments have been performed. To address one of the concerns noted above, we examined three different genomic regions associated with IL32 transcript variants and tested the primer-sets before we selected our final primer-set, but not to the extent the process was optimized.

As we were processing our data, we discovered a study by Park et al. [43] describing IL32 expression in MDA MB231 and MCF7. The investigators found that Interleukin 32 beta (IL32 β) stimulates both MDA MB231 and MCF cells and defined the mechanism as related to vascular endothelial growth factor- signal transducer and activator of transcription 3 (VEGF-STAT3) signaling. These data would suggest both cell lines have receptor molecules available for stimulation by IL32. Our experimental results repeatedly show that under normal growth conditions higher levels of IL32 in MDA MB231, when transcript levels detecting several variants are assessed. Our studies allowed for detection of a cocktail of IL32 transcript variants (including IL32 β); so the targets examined and the focus of their and our studies is very different.

Not all of the TNBC examined in our study were positive for IL32; some of the TMA cores were negative (even though CD31 activity was detected). In addition, a smaller portion of the non-TNBC tissues showed evidence of IL32 expression, while many of the normal tissues showed negligible levels. Related to the GSE34211 datasets, only 7 of the 18 triple negative cell lines showed substantial levels of IL32 expression compared to luminal subtype, while the MCF10A and MCF12A cell lines expressed IL32 levels comparable to that observed in the luminal subtype. Interestingly, five of the same cell lines (i.e., HCC1569, HCC1143, MDA MB436, Cal851 and Hs578T) demonstrated IL32 overexpression in both GSE34211 and GSE12777 datasets. Considering these data altogether, IL32 demonstrates a particular pattern of gene expression worthy of further consideration.

## Conclusion

We are aware that a limited number of datasets and samples are analyzed and processed for the current experiments. However as a preliminary study taking into account our results combined with information gathered from the GEO public datasets, the data suggest IL32 is differentially expressed in breast samples. It is important to both repeat and expand our studies and determine the implications of the IL32 gene expression pattern, using larger numbers of patient samples with extensive accompanying clinical data.

## Abbreviations

TNBC: Triple negative breast cancers; IL32: Interleukin 32; bp: Base pair; GEO: Gene expression omnibus; ESR: Estrogen receptor; PR: Progesterone receptor; TMA: Tissue microarray; CD31: Cluster of differentiation 1; MRPL19: Mitochondrial ribosomal protein L19; IHC: Immunohistochemistry; RefSeq: Reference sequences; IL6: Interleukin 6; LCK: Lymphocyte specific kinase; VEGF-STAT3: Vascular endothelial growth factor- signal transducer and activator of transcription 3; IL32 β: Interleukin beta; SOCS3: Suppressor of Cytokine Signaling 3 molecules.

## Competing interests

As a conflict of interest, AP is Associate Editor of the BMC Research Notes Journal. All other authors have no competing interests.

## Authors’ contributions

TO, KB and AP performed data analyses. AP, TO, JO and MH performed experiments. AP designed the study, analyzed the data and supervised the study. AP wrote the manuscript. All authors read and approved the final manuscript.

## Supplementary Material

Additional file 1: Table S1 Analysis of IL32 splice variants and comparison to current PCR primer-set. Table lists the RefSeq splice variants for the IL32 gene [16,17]. Yes/no refers to whether or not our IL32 primer-set recognizes the particular splice variant. The numbers in parenthesis represent the amino acids associated with the protein that differ from what is considered the canonical IL32 sequence. Click here for file

Additional file 2: Table S2 List of Ensembl protein and transcript identifications for the sequences used to generate the commercial IL32 antibody [15,21]. The table lists the protein and transcripts that correspond to the antigenic regions of the commercial IL32 antibody. The final column identifies the proteins/transcripts that are also recognized by the primers used for detecting our 207 bp PCR amplicon. Alignment based on Multialign™. Click here for file

Additional file 3: Figure S1 Ensembl transcripts and NCBI IL32 RefSeq splice variant comparisons to our 207 base amplicon. The 207 bp PCR amplicon is aligned with the Ensembl transcripts that match the epitope for the IL32 antibody and the IL32 RefSeq splice variants. The dashed arrows show the start to stop of the 207 bp amplicon nucleotides (approximately 576–783). Arrow designates the amplicon position above the consensus sequences. Red color shows concordance across all samples; blue color shows partial concordance. The PCR amplicon aligns with all but two RefSeq variants and three transcripts matching the protein epitope (circles). Click here for file

Additional file 4: Table S3 Summary of clinical samples on TMA. All of the designations were assigned by the vendor. The pathology, nature, stage, ER/PR/Her2 information was supplied by Pantomics Inc. ER/PR/Her2 analyses were performed by the vendor using IHC technique; signal intensities were also determined by the vendor. Clinical samples negative for ER/PR/Her2 are designated TNBC and highlighted in yellow. The CD31 and IL32 signal intensities were scored by 2 operators (AP and TO). The samples labeled with “**” were used for IHC figure 2. Click here for file
