# Supplementary material for: Preliminary characterization of IL32 in basal-like/triple negative compared to other types of breast cell lines and tissues
Source: BMC Res Notes. 2014 Aug 7;7:501. doi: 10.1186/1756-0500-7-501 (PMC4132244; doi:10.1186/1756-0500-7-501)
Supplement: Additional file 1: Table S1 — Analysis of IL32 splice variants and comparison to current PCR primer-set. Table lists the RefSeq splice variants for the IL32 gene [16,17]. Yes/no refers to whether or not our IL32 primer-set recognizes the particular splice variant. The numbers in parenthesis represent the amino acids associated with the protein that differ from what is considered the canonical IL32 sequence. [file 1756-0500-7-501-S1.docx]

**Additional file 1: Table S1- Analysis of IL32 splice variants and comparison to current PCR primer-sets.**

|  | |  | | |  | | | |  | | |
| --- | --- | --- | --- | --- | --- | --- | --- | --- | --- | --- | --- |
|  |  | |  | | |  | | | | |  |
| **Reference (NCBI)** | **Description** | | | **Description from GeneCards (corresponding protein)** | | | | **Primers recognize** | | |  |
| NM_001012631.1 | transcript variant 1 | | | canonical sequence | | | | yes | |  |  |
| NM_004221.4 | transcript variant 2 | | | Beta (19-64) | | | | yes | |  |  |
| NM_001012632.1 | trascript variant 3 | | | Isoform B (1-10 ; 19-64 ) | | | | yes | |  |  |
| NM_001012633.1 | transcript variant 4 | | | Alpha (19-64 ; 154-210) | | | | no | |  |  |
| NM_001012634.1 | transcript variant 5 | | | Isoform C (19-64 ; 65-84) | | | | yes | |  |  |
| NM_001012635.1 | transcript variant 6 | | | Isoform C (19-64 ; 85-93) | | | | yes | |  |  |
| NM_001012636.1 | transcript variant 7 | | | Isoform D (19-84 ; 94-113) | | | | no | |  |  |
| NM_001012718.1 | transcript variant 8 | | | Beta (same as 1) | | | | yes | |  |  |
|  |  | |  | | | |  | | | |  |
|  |  | |  | | | |  | | | |  |
|  |  | |  | | | |  | | | |  |
|  |  | |  | | | |  | | | |  |
|  |  | |  | | | |  | | | |  |
|  |  | |  | | | |  | | | |  |
|  |  | |  | | | |  | | | |  |
