# Supplementary material for: Preliminary characterization of IL32 in basal-like/triple negative compared to other types of breast cell lines and tissues
Source: BMC Res Notes. 2014 Aug 7;7:501. doi: 10.1186/1756-0500-7-501 (PMC4132244; doi:10.1186/1756-0500-7-501)
Supplement: Additional file 2: Table S2 — List of Ensembl protein and transcript identifications for the sequences used to generate the commercial IL32 antibody [15,21]. The table lists the protein and transcripts that correspond to the antigenic regions of the commercial IL32 antibody. The final column identifies the proteins/transcripts that are also recognized by the primers used for detecting our 207 bp PCR amplicon. Alignment based on Multialign™. [file 1756-0500-7-501-S2.docx]

| **Additional file 2: Table S2:**  **List of Ensembl protein and transcript identifications for the sequences used to generate the commercial IL32 antibody** | | | | | | | | | |
| --- | --- | --- | --- | --- | --- | --- | --- | --- | --- |
|  | | | | |  |  |  |  |  |
|  |  |  |  |  |  |  |  |  |  |
| Antigen | Recombinant protein fragment | |  |  |  |  |  |  |  |
| Length (aa) | 81 |  |  |  |  |  |  |  |  |
| Antigen sequence | FPKVLSDDMKKLKARMHQAIERFYDKMQNAESGRGQVMSSLAELEDDFKE | | | | | | |  |  |
|  | GYLETVAAYYEEQHPELTPLLEKERDGLRCR | | | | | | |  |  |
|  |  |  |  |  |  |  |  |  |  |
|  | **IL32 transcript / % matching with antigen sequence** | | | | |  | Align with |  |  |
|  |  | Protein ID |  |  | Transcript ID |  | PCR amplicon |  |  |
| **Matching transcripts** | IL32-001 - ENSP00000324742 [100%] | | | | [ENST00000325568](http://sep2013.archive.ensembl.org/Homo_sapiens/Transcript/Summary?db=core;g=ENSG00000008517;r=16:3115298-3119668;t=ENST00000325568) |  | yes |  |  |
|  | IL32-004 - ENSP00000380096 [100%] | | | | [ENST00000396887](http://sep2013.archive.ensembl.org/Homo_sapiens/Transcript/Summary?db=core;g=ENSG00000008517;r=16:3115298-3119668;t=ENST00000396887) |  | no |  |  |
|  | IL32-008 - ENSP00000432218 [100%] | | | | [ENST00000525643](http://sep2013.archive.ensembl.org/Homo_sapiens/Transcript/Summary?db=core;g=ENSG00000008517;r=16:3115298-3119668;t=ENST00000525643) |  | yes |  |  |
|  | IL32-009 - ENSP00000411958 [100%] | | | | [ENST00000444393](http://sep2013.archive.ensembl.org/Homo_sapiens/Transcript/Summary?db=core;g=ENSG00000008517;r=16:3115298-3119668;t=ENST00000444393) |  | yes |  |  |
|  | IL32-012 - ENSP00000437020 [100%] | | | | [ENST00000529550](http://sep2013.archive.ensembl.org/Homo_sapiens/Transcript/Summary?db=core;g=ENSG00000008517;r=16:3115298-3119668;t=ENST00000529550) |  | yes |  |  |
|  | IL32-013 - ENSP00000436929 [100%] | | | | [ENST00000530538](http://sep2013.archive.ensembl.org/Homo_sapiens/Transcript/Summary?db=core;g=ENSG00000008517;r=16:3115298-3119668;t=ENST00000530538) |  | yes |  |  |
|  | IL32-014 - ENSP00000432917 [100%] | | | | [ENST00000533097](http://sep2013.archive.ensembl.org/Homo_sapiens/Transcript/Summary?db=core;g=ENSG00000008517;r=16:3115298-3119668;t=ENST00000533097) |  | yes |  |  |
|  | IL32-022 - ENSP00000450364 [100%] | | | | [ENST00000526464](http://sep2013.archive.ensembl.org/Homo_sapiens/Transcript/Summary?db=core;g=ENSG00000008517;r=16:3115298-3119668;t=ENST00000526464) |  | yes |  |  |
|  | IL32-024 - ENSP00000405063 [100%] | | | | [ENST00000440815](http://sep2013.archive.ensembl.org/Homo_sapiens/Transcript/Summary?db=core;g=ENSG00000008517;r=16:3115298-3119668;t=ENST00000440815) |  | yes |  |  |
|  | IL32-025 - ENSP00000447496 [100%] | | | | [ENST00000551122](http://sep2013.archive.ensembl.org/Homo_sapiens/Transcript/Summary?db=core;g=ENSG00000008517;r=16:3115298-3119668;t=ENST00000551122) |  | no |  |  |
|  | IL32-027 - ENSP00000432850 [100%] | | | | [ENST00000528163](http://sep2013.archive.ensembl.org/Homo_sapiens/Transcript/Summary?db=core;g=ENSG00000008517;r=16:3115298-3119668;t=ENST00000528163) |  | yes |  |  |
|  | IL32-028 - ENSP00000447812 [100%] | | | | [ENST00000549213](http://sep2013.archive.ensembl.org/Homo_sapiens/Transcript/Summary?db=core;g=ENSG00000008517;r=16:3115298-3119668;t=ENST00000549213) |  | no |  |  |
|  | IL32-031 - ENSP00000448683 [100%] | | | | [ENST00000552664](http://sep2013.archive.ensembl.org/Homo_sapiens/Transcript/Summary?db=core;g=ENSG00000008517;r=16:3115298-3119668;t=ENST00000552664) |  | yes |  |  |
|  |  |  |  |  |  |  |  |  |  |
|  |  |  |  |  |  |  |  |  |  |
|  |  |  |  |  |  |  |  |  |  |
