# Supplementary figures and images for: Preliminary characterization of IL32 in basal-like/triple negative compared to other types of breast cell lines and tissues
Source: BMC Res Notes. 2014 Aug 7;7:501. doi: 10.1186/1756-0500-7-501 (PMC4132244; doi:10.1186/1756-0500-7-501)

Supplemental figure 1-Ensembl transcripts and NCBI RefSeq comparisons to IL32 PCR amplicon

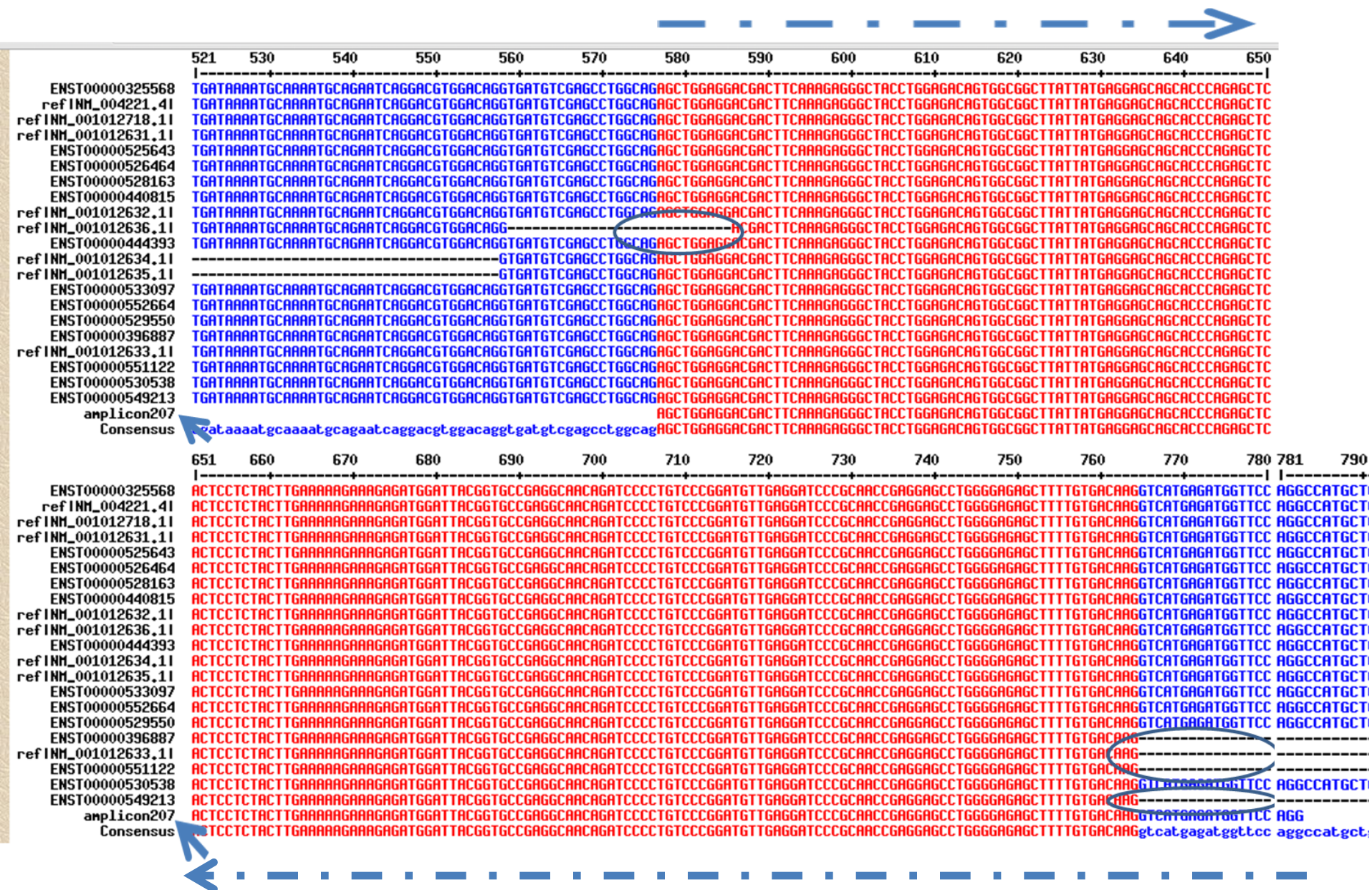

Supplement: Additional file 3: Figure S1 — Ensembl transcripts and NCBI IL32 RefSeq splice variant comparisons to our 207 base amplicon. The 207 bp PCR amplicon is aligned with the Ensembl transcripts that match the epitope for the IL32 antibody and the IL32 RefSeq splice variants. The dashed arrows show the start to stop of the 207 bp amplicon nucleotides (approximately 576–783). Arrow designates the amplicon position above the consensus sequences. Red color shows concordance across all samples; blue color shows partial concordance. The PCR amplicon aligns with all but two RefSeq variants and three transcripts matching the protein epitope (circles). [file 1756-0500-7-501-S3.pdf]
